# A Geographical Approach to China’s Local Government Debt

**DOI:** 10.1080/00330124.2023.2300803

**Published:** 2024-03-04

**Authors:** Zhenfa Li, Fulong Wu, Fangzhu Zhang

**Affiliations:** University College London, UK

**Keywords:** Chengtou bonds, China, intergovernmental relations, local government bonds, local government debt, 城投债券, 中国, 政府间关系, 地方政府债券, 地方政府债务, bonos Chengtou, bonos de gobiernos locales, China, deuda del gobierno local, relaciones intergubernamentales

## Abstract

Since the 2010s local government debt has boomed in China because the government relies on debt financing for infrastructure investment. The debt mainly consists of the issuance of *Chengtou* bonds and later local government bonds. Using data from more than 300 cities from 2009 to 2020, this article maps its spatial dynamics to further the understanding of intergovernmental relations in the studies on local government debt. We find that, from 2009 to 2014, most cities had large bond-issuing amounts. The dynamics were affected by the economic stimulus target set by the central government and the interjurisdictional competition in borrowing among local governments. After 2015 the cities with better economies issued more bonds because the central government tried to match local government debt with local fiscal capacity to maintain financial security. The spatial dynamics show the increasing intervention by the central government in local fiscal income and expenditure, reflecting fiscal centralization. Fiscal centralization did not effectively contain the financial risk in the less-developed cities. Motivated by the competition, the less-developed cities did not use bonds efficiently and had higher ratios of bond issuance to fiscal income, experiencing higher financial risk.

## Abstract (zh)

自2010年代以来, 由于中国地方政府通过债务融资进行基础设施投资, 地方政府债务激增。债务主要包括城投债券以及后来的地方政府债券。本文利用2009年至2020年300多个城市的数据, 绘制了空间变化图, 进一步了解地方政府债务研究中的政府间关系。我们发现, 从2009年到2014年, 多数城市都发行了大量的债券。这种变化受到中央政府的经济刺激目标和地方政府之间跨域借贷竞争的影响。2015年后, 为了维持金融安全, 中央政府试图将地方政府债务与地方财政能力相匹配。因此, 经济状况良好的城市发行了更多的债券。空间变化显示, 中央政府对地方财政收支的干预持续增加, 这体现了财政集中化。财政集中化并未有效遏制欠发达城市的金融风险。在竞争的推动下, 欠发达城市未能有效地利用债券, 债券发行与财政收入的比例更高, 从而面临着更高的金融风险。

## Abstract (es)

Desde el 2010, la deuda de los gobiernos locales en China se ha disparado debido a que las administraciones recurren a la financiación por medio de deuda para inversión en infraestructura. La deuda consiste primordialmente en la emisión de bonos Chengtou y, más tarde, bonos del gobierno local. Usando datos de más de 300 ciudades, entre 2009 y 2020, este artículo mapea la dinámica espacial para lograr un mayor entendimiento de las relaciones intergubernamentales en los estudios sobre deuda de los gobiernos locales. Encontramos que, de 2009 a 2014, la mayoría de las ciudades emitieron grandes volúmenes de bonos. Las dinámicas se afectaron por el objetivo del estímulo económico establecido por el gobierno central y por la competencia interjurisdiccional en el endeudamiento entre los gobiernos locales. Después de 2015, las ciudades con mejores economías emitieron más bonos debido a que el gobierno central trató de equiparar la deuda del gobierno local con la capacidad fiscal local para conservar la seguridad financiera. La dinámica espacial deja ver la creciente intervención del gobierno central en los ingresos y gastos fiscales locales, lo cual refleja la centralización fiscal. La centralización fiscal no contuvo eficazmente el riesgo financiero en las ciudades menos desarrolladas. Motivadas por la competencia, las ciudades menos desarrolladas no utilizaron eficientemente los bonos y tuvieron ratios más altas de emisión de bonos respecto a los ingresos fiscales, experimentando un riesgo financiero más alto.

China has seen large-scale infrastructure construction. The debt financing for infrastructure investment led by the government results in surging local government debt and financial risk (Pan et al. 2017; Z. Li, Wu, and Zhang 2022; Liu, Oi, and Zhang 2022). The debt mainly comes from local government financing platforms (LGFPs) that borrow from banks and issue corporate bonds (known as *Chengtou* bonds) and then local government bonds (LGBs) issued by provincial governments. By the end of 2022, the balance of outstanding LGBs was 35 trillion Yuan and that of *Chengtou* bonds was 15 trillion Yuan. Existing studies have examined LGFPs and LGBs (Feng, Wu, and Zhang 2022; Z. Li, Wu, and Zhang 2022; Ye et al. 2022), but few use a geographical approach to examine local government debt in different stages. This article investigates the city-level spatial dynamics of local government debt from 2009 to 2020 and hopes to make the following contributions.

Theoretically, the spatial dynamics improve the understanding of intergovernmental relations that shape local government debt. The dynamics resulted from the central policies of stimulating economic growth and then restricting government debt, and local governments’ pursuit of political career promotion also showed an effect. The dynamics show the increasing intervention by the central government in local fiscal income and expenditure, reflecting fiscal centralization.

The dynamics show that the less developed cities did not use *Chengtou* bonds and LGBs efficiently and had higher financial risk. These findings extend the understanding of fiscal centralization, which did not effectively contain the disproportionately higher risk experienced by the less developed cities.

Methodologically, the dynamics are a mesolevel method different from an aggregated analysis at the national level or a case study based on specific cities. Based on a disaggregated geographical perspective, this method better examines the general situation of local government debt in China by showing the differences and similarities of cities across the country and advances the understanding of fiscal centralization. The findings on the uneven distribution of financial risk generate practical political economic implications for the central and local governments, posing a tricky question about how to alleviate the risk.

Empirically, this article shows the most recent city-level geography of local government debt in China from 2009 to 2020. To the best of our knowledge, this is the first attempt to describe the patterns within this time frame.

## Local Government Debt and Intergovernmental Relations in China

### Local Government Debt

The literature mainly examines the debt of LGFPs (Tsui 2011; Pan et al. 2017). To counter the global financial crisis in 2008, the State Council initiated an economic stimulus package worth 4 trillion Yuan (Naughton 2009). The package required local governments to raise more than 2 trillion Yuan for infrastructure investment by the end of 2010 to maintain economic growth. Local governments, mainly county- and city-level governments, set up LGFPs as local state-owned enterprises that borrowed from the market for infrastructure construction (Feng, Wu, and Zhang 2022). LGFPs mainly borrowed bank loans and issued corporate bonds (known as *Chengtou* bonds). To maximize the financing capacity of LGFPs, local governments injected the use right of land into the platforms as collateral to loans and bond issuance (Wu 2022).

Some LGFP debt was counted as local government debt because the local government sometimes gave payment guarantees to the investors, promising that they would pay LGFP debt if the companies could not do so. The guarantees were not made public but only known between a few officials and investors. Such opacity made some LGFP debt “implicit local government debt” that local governments might be responsible for repaying (Z. Li, Wu, and Zhang 2022). The platforms had limited revenue streams due to their main functions and could hardly pay the debt, and local fiscal income was also far from enough. LGFPs could only borrow more to repay during and after the package. As a result, local government debt surged and led to financial risk.

To manage the financial risk, the central government promoted LGBs in 2015 to replace LGFPs as the main financing source for infrastructure investment. LGBs have unique features shaped by the political economic context of China (Z. Li, Wu, and Zhang 2023a, 2023b). The provincial government issues and repays LGBs for itself and on behalf of the lower level governments. It transfers money to and collects repayment from the lower level governments. From 2015 to 2018, the central government used LGBs to swap the “implicit local government debt” accumulated by the end of 2014, which meant that the debtors replaced LGFP debt guaranteed by local governments with an equal amount of LGBs. Local government debt has become more transparent, and the more fiscally powerful provincial government could afford the debt that was excessive for county- and city-level governments.

LGBs are divided into general bonds and special bonds. General bonds finance projects without revenue streams and the repayment comes from fiscal income. Special bonds are for projects with yields and are paid mostly by future income of the infrastructure. The central government sets annual bond quotas, and local governments at different levels apply for their quotas before they can issue bonds or use bond capital. The applications are submitted to the next upper level government first and finally to the Ministry of Finance. The Ministry evaluates the applications and then calculates quotas. The Ministry’s top priority in calculating quotas is to match local government debt and local fiscal capacity. Meanwhile, in late 2014 the State Council prohibited local governments from issuing payment guarantees and thus separated LGFP debt from local government debt. LGFPs still finance infrastructure investment but act as local state-owned enterprises based on their financial capacity. By doing these, the central government has tried to make local government debt more sustainable to restrict financial risk.

### Intergovernmental Relations

Existing studies tend to examine local government debt in China from the perspective of intergovernmental relations between the central and local governments and between local governments at the same administrative level. This article has the same analytical focus but hopes to produce new insights into how changing intergovernmental relations shape local government debt.

Many studies examine the influence of central–local fiscal relations on local fiscal income and expenditure (Huang 1996; Zhang 1999; Wong 2000). After the economic reform in 1978, the State Council initiated a “fiscal contracting system” to devolve fiscal authority to provinces. Local governments submitted a certain amount of fiscal income and kept the rest for expenditure. This system incentivized local governments to promote development and increase fiscal income compared to the earlier planned economic system in which the central government allocated economic resources. Nonetheless, the ratio of central fiscal income to national fiscal income decreased to less than 30 percent in the early 1990s. The State Council enacted a tax reform in 1994 that enabled the central government to collect a large proportion of local tax income and leave most expenditure tasks to local governments. The authority over fiscal income was centralized but that over fiscal expenditure remained decentralized. After the reform, local governments started to experience a widening gap between income and expenditure (Zhan 2013). Meanwhile, the Budget Law in 1994 stipulated that local governments could not directly borrow from the market.^1^ Local governments started to rely on “land finance” in the late 1990s (Cao, Feng, and Tao 2008). The income from leasing the use right of land was not collected by the central government and became the most important source of local fiscal income.

Other studies pay attention to the relations between local governments at the same administrative level, mainly the interjurisdictional competition. They believe that local officials tend to compete with their peers in driving economic growth and increasing fiscal income, particularly through land finance because better economic performance is key to local official promotion (Qun, Li, and Yan 2015; He, Zhou, and Huang 2016). They mostly draw on a “promotion tournament model” suggesting that local officials achieving better economic development are more likely to be promoted by the upper level decision-makers who emphasize economic growth (H. Li and Zhou 2005; Zhou 2016). Aligning with the central government’s objectives not only in growth but also in other aspects such as rural revitalization, industrial upgrade, and environmental protection is also key to promotion (Wu, Zhang, and Liu 2022).

These perspectives are used to explain local government debt. The stimulus package was only a catalyst for the establishment of LGFPs. These platforms resulted from the long-standing fiscal shortage faced by local governments (Liu, Oi, and Zhang 2022). Land finance created large income but could not cope with the stimulus target, and there was interjurisdictional competition in establishing LGFPs (Pan et al. 2017).

Few studies, however, discuss LGFPs from a centralization–decentralization perspective, although Z. Li, Wu, and Zhang (2023b) suggested that LGBs reflect fiscal centralization in income and expenditure after the tax reform in 1994 due to the quota system.

### Advancing the Understanding by a Geographical Approach

The understanding of intergovernmental relations in local government debt could be furthered. LGFPs, LGBs, and the shift from LGFPs to LGBs reveal analytically significant nuances of changing intergovernmental relations regarding fiscal decentralization and centralization. The nuances can hardly be examined at the national level. For example, Z. Li, Wu, and Zhang’s (2023b) conclusion about LGBs reflecting fiscal centralization comes from an examination of the quota system applied to the whole country and could be further elaborated. We identify three stages, including the periods of land finance (before 2009), LGFPs (2009–2014), and LGBs (after 2015), respectively. The quota system signaled the centralization of local fiscal income and expenditure compared to both the periods of land finance and LGFPs. Meanwhile, the similar fiscal centralization happened in the period of LGFPs compared to the period of land finance.

Z. Li, Wu, and Zhang (2023b) mentioned the financial risk caused by the large issuance of LGBs. The risk also came, however, from LGFPs, as local governments still relied on *Chengtou* bonds as infrastructure finance after 2015. More important, the risk was unevenly distributed, and the less developed cities experienced higher risk due to limited fiscal capacity. Fiscal centralization did not constrain the local governments’ reliance on debt financing and consequently could not contain the disproportionately higher risk experienced by the less developed cities.

Such nuances can hardly be revealed by case studies, either, as their potential is restricted by local specificities. Whether conclusions produced by case studies could be generalized to the country is questionable, especially in a country like China with a salient regional disparity in economic development and government fiscal capacity.

This article uses a mesolevel method that investigates the city-level spatial dynamics to show the nuances. The dynamics indicate both nationwide and local situations by showing the features of every city and the relations between cities. The findings produced by the dynamics advance the understanding of fiscal centralization and have practical political economic implications because they reveal an urgent problem for the central and local governments.

The mesolevel method makes contributions beyond China in terms of how disaggregated spatial dynamics can be used to examine intergovernmental relations. Scholars who examine local government debt in different contexts tended to focus on national features or local specificities but have recently called for a geographically disaggregated perspective to better understand debt (e.g., Psycharis, Zoi, and Iliopoulou 2016; Davidson, Lukens, and Ward 2021; Dagdeviren and Karwowski 2022). We respond to the call and extend the existing understanding with the perspectives generated in the Chinese context.

## Data and Methodology

### Data

This article examines the city-level spatial dynamics of LGFPs, LGBs, and the shift from LGFPs to LGBs from 2009 to 2020 using two data sets. We use the issuance of *Chengtou* bonds to represent the debt of LGFPs, as the data on bank loans are never disclosed. The data from 2009 to 2020 are downloaded from the WIND database, an authoritative third-party financial database in China. The data on LGB issuance from 2015 to 2020 are manually created by retrieving more than 20,000 disclosed reports on China Central Depository & Clearing platform and are original.

LGBs have different categories. We select newly issued special bonds used for the new financing requirements from new or ongoing profitable infrastructure projects to represent LGBs. This category accounts for the major part of LGBs, and the platform publishes disclosed reports on infrastructure projects using this category. The information includes the volume of bonds used and the project’s location. Other supplementary data, such as population and gross domestic product (GDP) per capita, come from statistical yearbooks of China. News related to local government debt from reliable news agencies are used to support the findings and explain the political economic implications.

### Method

This article produces city-level thematic maps and calculates statistics using ArcGIS. The statistics include global Moran’s I, Getis–Ord general G, and Anselin local Moran’s I. These statistics reflect whether the specific value (bond issuance) of the features (cities) is clustered or randomly distributed in the spatial pattern (city-level spatial dynamics of local government debt).^2^

Global Moran’s I measures spatial autocorrelation by feature locations and values (Goodchild et al. 2000). The null hypothesis is that the feature values are spatially uncorrelated, which remains the same for the following statistics. If it is rejected (*z* score is less than −1.65 or greater than 1.65 and *p* value is between 0 and 0.1 at a significance level of 0.1, and this range applies to the following statistics), a positive index suggests a tendency of clustering. A negative index means a tendency of dispersion. If the null hypothesis cannot be rejected, the feature values tend to be randomly distributed.

Getis–Ord general G measures the degree of clustering for high or low feature values (Getis and Ord 2010). There are high- or low-value clusters if the null hypothesis is rejected. If the *z* score is less than −1.65, there are clusters of low feature values, whereas the *z* score being greater than 1.65 means the clustering of high feature values.

Anselin’s local Moran’s I produces thematic maps to indicate the locations of clusters with different characteristics (Anselin 1995). If the *z* score is greater than 1.65, the feature is surrounded by features with similar values. The clusters with similarly high values are categorized as high–high clusters, whereas those with similarly low values are low–low clusters. If the *z* score is less than −1.65, the feature is surrounded by features with significantly different values. A high-value feature being surrounded by low-value features is named high–low outliers. Otherwise, it is low–high outliers. When calculating these statistics, we select “inverse distance” to define the spatial relationship between feature values.

The clustering analysis supports the examination of fiscal centralization. The emergence and disappearance of clustering tendency and the clusters with different features in different stages resulted from the dynamic intergovernmental relations. Moreover, the clustering analysis reveals the influence of the investors in *Chengtou* bonds and LGBs over the spatial pattern. The investors considered the fiscal and economic conditions of a city, and they were affected by government policies and objectives because they were mostly state-owned commercial banks.

## The Spatial Dynamics from 2009 to 2014

**Figure 1:**
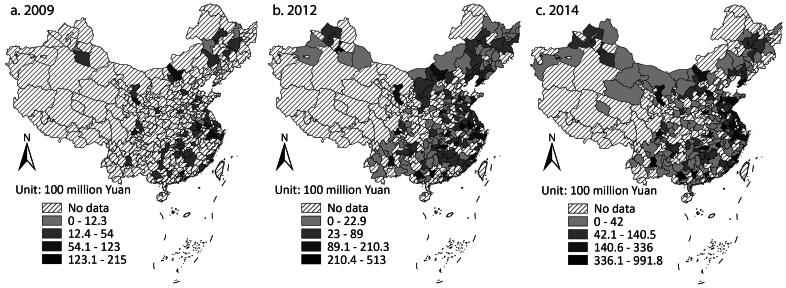
City-level Chengtou bond issuance in (A) 2009, (B) 2012, and (C) 2014.

Figure 1 shows the spatial dynamics in this period. The cities in the east issued more *Chengtou* bonds.^3^ Many cities in the middle, western, and northeast regions also had large issuance, however. There were some cities with very large amounts scattered in these regions. Most of them were provincial capital cities with strong economies, such as Lanzhou in Gansu Province, Changsha in Hunan, Chengdu in Sichuan, Kunming in Yunnan, and so on. The provincial capital cities in the east also issued more bonds than other cities in the province.

**Figure 2:**
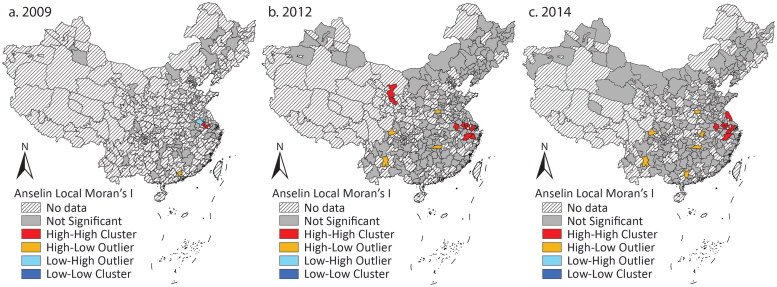
Clusters and outliers of Chengtou bond issuance in (A) 2009, (B) 2012, and (C) 2014.

Table 1 shows that, in general, there was no tendency for clustering. In this period, more and more cities experienced rapid growth in *Chengtou* bond issuance, which narrowed the intercity gap and constrained the tendency of high-value clustering. Figure 2 demonstrates that the high–high clusters and the high–low outliers corresponded to the cities in the east and the provincial capital cities with large issuance.

**Table 1:** Global Moran’s I and Getis–Ord general G of Chengtou bond issuance in 2009, 2012, and 2014

	2009			2012			2014		
	**Index**	***Z* score**	***p* value**	**Index**	***Z* score**	***p* value**	**Index**	***Z* score**	***p* value**
Global Moran’s I	−0.038	−0.331	0.741	0.064	1.828	0.068	0.003	0.259	0.795
Getis–Ord general G	0.017	1.004	0.315	0.006	2.015	0.044	0.005	0.831	0.406

The dynamics show that the cities in some eastern provinces and provincial capital cities with better economies issued more bonds. Understandably, the more developed cities had larger demand for infrastructure investment and better fiscal capacity to pay the debt. Many less developed cities also had large issuance, though. Due to the pressure from the stimulus package and the subsequent repayment, cities across the country had to keep borrowing through *Chengtou* bonds. Local officials were keen to achieve the stimulus target, as it was emphasized by the central government. The central government even published a policy encouraging local governments to establish LGFPs to expand infrastructure investment by borrowing from the financial market.^4^ In contrast, debt repayment was not a priority at that time. Meanwhile, Naughton (2009) suggested that local officials regarded the stimulus package as an opportunity to drive economic growth because they could start many projects they could not afford without the stimulus package. Provinces competed in starting projects. According to the state media Xinhua, every province had sent hundreds of projects to the Ministry of Finance for review by November 2008, just one month after the stimulus package was initiated.^5^ In summary, the distribution was mainly determined by the stimulus package set by the central government, and the local officials’ pursuit of career promotion motivated them to achieve the stimulus target and undertake more projects to drive growth, reinforcing the pattern that many cities had large bond issuance.

Local governments in the less developed regions could borrow heavily because the investors were willing to invest in these regions. The investors were mostly central and local state-owned commercial banks (Z. Li, Wu, and Zhang 2023a). Their majority shareholders are central or local government departments or state-owned enterprises that decide the appointments of senior positions. Therefore, they usually support government objectives (Firth, Lin, and Wong 2008). Naughton (2009) mentioned that bank managers tended to invest in LGFPs because they were evaluated by how well they supported the stimulus target rather than how efficient their investment decisions were. On the other hand, they considered a return on investments. Multiple media reports suggested that, as land prices increased rapidly nationwide, they were willing to invest in LGFPs with land use right as collateral to seek long-term and high rates of return.^6^ The banks also deemed investing in LGFPs a safe option due to the payment guarantees from local governments. The debt might exceed local fiscal capacity, but the investors believed that the government would not actually default on its debt. These considerations made the dynamics mainly decided by the government, as the investors were likely to purchase *Chengtou* bonds issued regardless of the locations.

The dynamics furthered the understanding of intergovernmental relations in the early 2010s. As mentioned earlier, few studies examined LGFPs from a centralization–decentralization perspective. In the period of land finance, the central government collected a large proportion of local fiscal income but hardly intervened in how local governments raised money for expenditure. In this period, the central government actively encouraged LGFPs to borrow and encouraged the banks to lend. It might be inappropriate to say that the authority over local fiscal income was centralized due to such instructions, but local governments indeed received more intervention from the central government in generating income by debt financing. For expenditure, the stimulus package revealed fiscal centralization because the central government directly affected how local governments spent their money by asking them to invest in infrastructure construction and setting key fields they should prioritize. Projects were sent to the central government for review before started (Naughton 2009).

## The Spatial Dynamics after 2015

### The Issuance of Chengtou Bonds and LGBs

**Figure 3:**
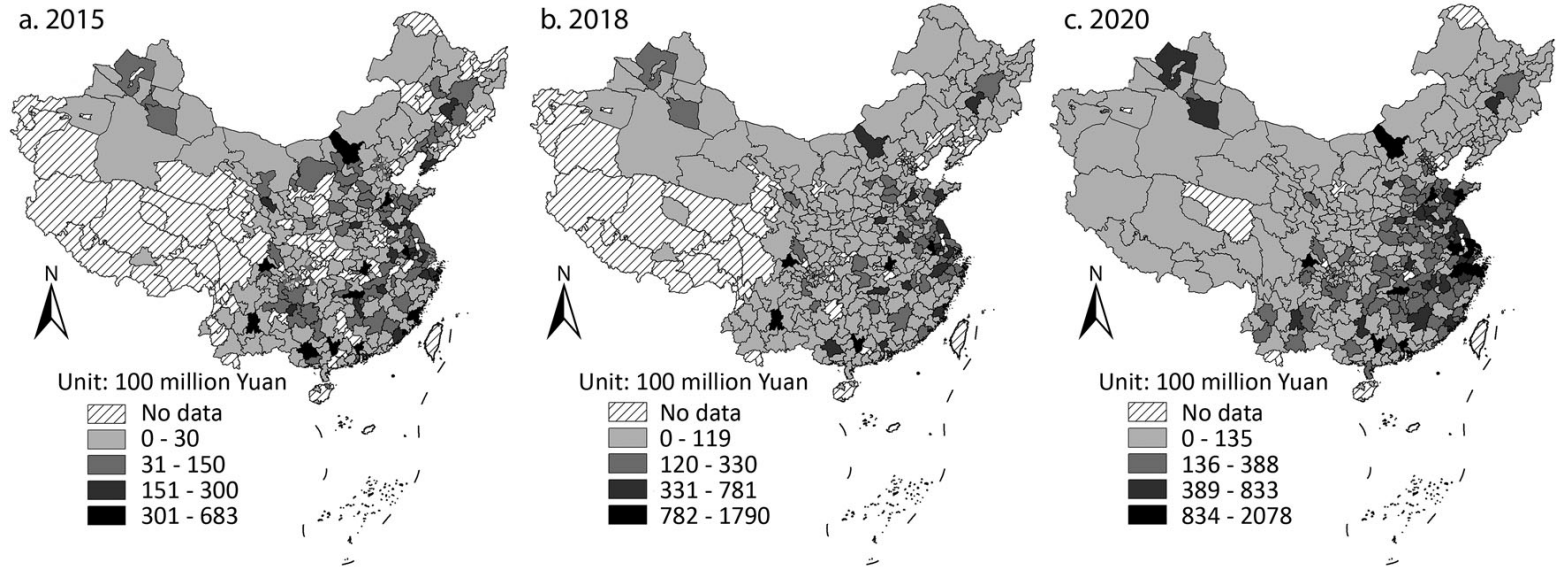
City-level issuance of Chengtou bonds and LGBs in (A) 2015, (B) 2018, and (C) 2020.

Although LGFPs independently finance infrastructure investment after 2014, scholars believe local governments will not let them default on their debt, as infrastructure construction in China is deemed a government effort and achievement. More important, local governments sometimes still issue payment guarantees secretly. LGFP debt is still local government debt in a broad sense (Pan et al. 2017; Liu, Oi, and Zhang 2022; Z. Li, Wu, and Zhang 2023b). Then, we use the combined amount of *Chengtou* bonds and LGBs to represent local government debt. Figure 3 shows the spatial dynamics. Bond issuance significantly increased across the country. The cities in the east issued most bonds, followed by the middle and then the west and northeast. The cities in the same province or region had similar issuing amounts. Provincial capital cities still issued more than their neighbors, but the gap was narrowed compared with the previous period.

**Figure 4:**
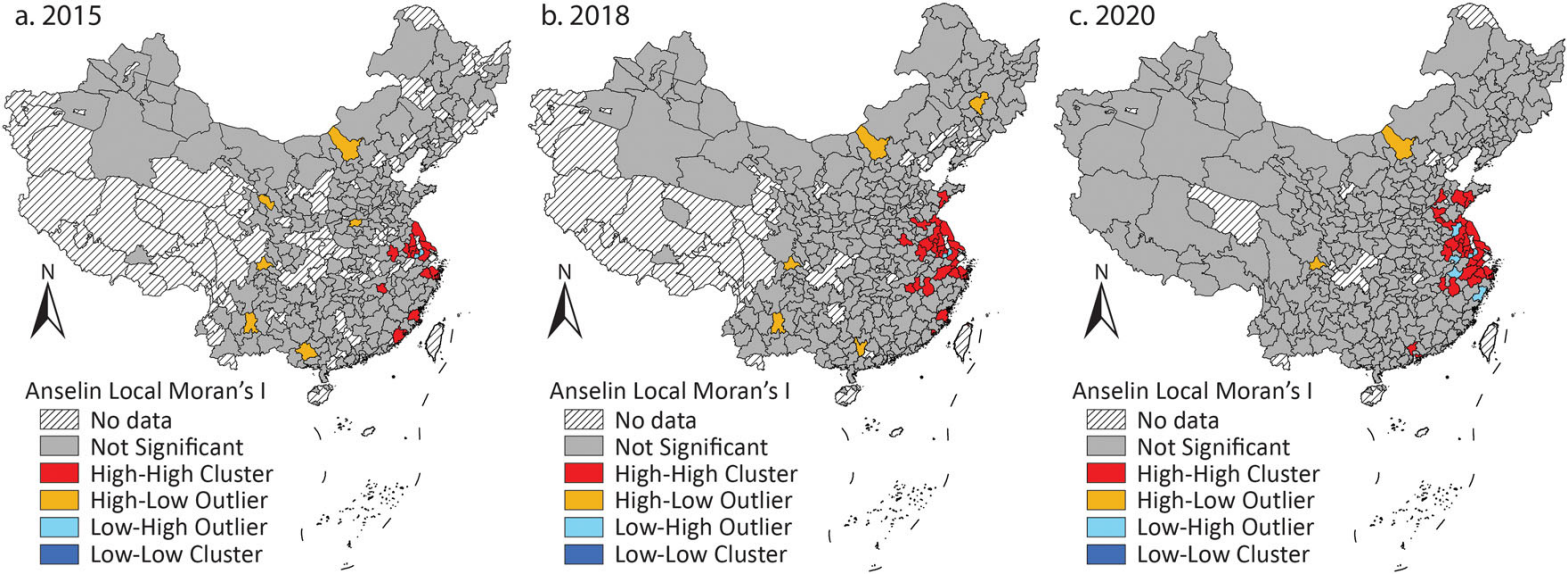
Clusters and outliers of the issuance of Chengtou bonds and local government bonds in (A) 2015, (B) 2018, and (C) 2020.

According to Table 2, there was a tendency to cluster cities with large issuance. Figure 4 shows that the high–high clusters were more than in the previous period and diffused into the middle. The high–low outliers, which mainly reflected the larger issuance of provincial capital cities than others, existed in 2015 and 2018 but almost disappeared in 2020. This could be explained by an increase in bond issuance by most cities. The gap between provincial capital cities and their neighbors gradually narrowed and could not reach the statistical significance to foster high–low outliers.

**Table 2:** Global Moran’s I and Getis–Ord general G of the issuance of Chengtou bonds and local government bonds in 2015, 2018, and 2020

	2015			2018			2020		
	Index	*Z* score	*p* value	Index	*Z* score	*p* value	Index	*Z* score	*p* value
Global Moran’s I	0.007	0.842	0.400	0.052	5.543	0.000	0.165	9.404	0.000
Getis–Ord general G	0.004	1.306	0.192	0.004	4.683	0.000	0.004	9.115	0.000

The dynamics reveal the Chinese government’s increasing reliance on debt financing for infrastructure investment. The debt accumulation was more regulated, though. In the previous period, cities across the country had large issuance of *Chengtou* bonds. After 2015, cities with better economies issued more bonds and fostered clusters. In addition to the quota system that matches local government debt and local fiscal income, the central government follows a “positive incentive” in allocating quotas. In 2017 an official in the Ministry of Finance suggested that the Ministry would allocate larger quotas to the regions with more fiscal and economic resources and lower debt ratios.^7^

The dynamics did not mean decreased local governments’ competition in borrowing. Local officials still tried to propose more projects to apply for LGB quotas. The central government promoted income-expenditure balanced newly issued special bonds in 2017 that required infrastructure to generate enough income in the future to pay investors with principal and interest. In 2020 the National Debt Association of China disclosed infractions conducted by local governments in quota applications, indicating that local governments sometimes exaggerated future income to make projects without enough revenue streams financed by the bonds.^8^ Such misconduct reflected local governments’ continuous enthusiasm for borrowing more. Nonetheless, the dynamics show that the competition was secondary to the regulations of the central government.

The investors also contributed to the dynamics. They purchased all the LGBs issued according to the disclosed reports but started to be more skeptical toward *Chengtou* bonds in the less developed regions. Z. Li, Wu, and Zhang (2023a) discussed why state-owned commercial banks change their investment priority from *Chengtou* bonds to LGBs. After the ban on local governments providing payment guarantees, the banks could not hold local governments accountable if LGFPs really default on their debt. From a political perspective, they did not need to support LGFPs as they had done when the support had been key to the stimulus target. This article shows that the skepticism was more likely to happen in the less developed regions.

The dynamics reflect the centralization of local fiscal income and expenditure compared to the period of LGFPs. The quota system and the positive incentive largely shaped the spatial dynamics and decided how much income local governments could create and how much money they could spend through bond issuance. In the period of LGFPs, the central government encouraged local governments to borrow and spend more, whereas in this period, the central government intervened more by setting specific issuing amounts of LGBs. Local governments were motivated to align with the quota system but still tended to borrow more to compete (Z. Li, Wu, and Zhang 2022). Nonetheless, their intention to compete did not affect the dynamics as significantly as the central government’s objective of restricting debt.

### The Shift from Chengtou Bonds to LGBs

**Figure 5:**
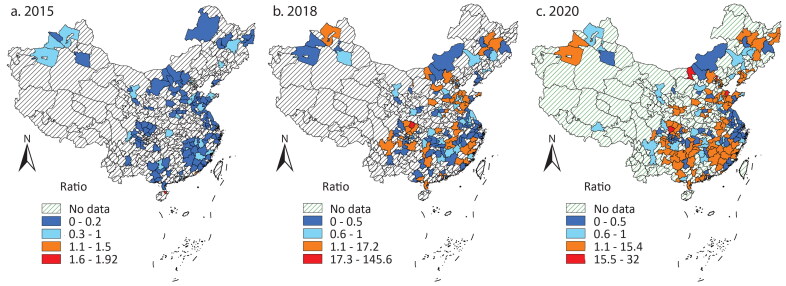
The ratio of local government bonds issuance to Chengtou bond issuance in (A) 2015, (B) 2018, and (C) 2020.

Figure 5 shows the spatial dynamics of the shift from *Chengtou* bonds to LGBs after 2015 by calculating the ratio of LGB issuance to *Chengtou* bond issuance by a city. Figure 5 shows that, first, the shift from *Chengtou* bonds to LGBs was a gradual process. In 2015, most cities issued more *Chengtou* bonds than LGBs. Then, more and more cities issued more LGBs than *Chengtou* bonds, and in 2020, more cities prioritized the use of LGBs over *Chengtou* bonds. Second, the less developed cities in the middle, west, and northeast regions relied more on LGBs than *Chengtou* bonds. Well-off cities in the east kept issuing more *Chengtou* bonds. Third, once a city switched from *Chengtou* bonds to LGBs, it rarely changed back.

**Figure 6:**
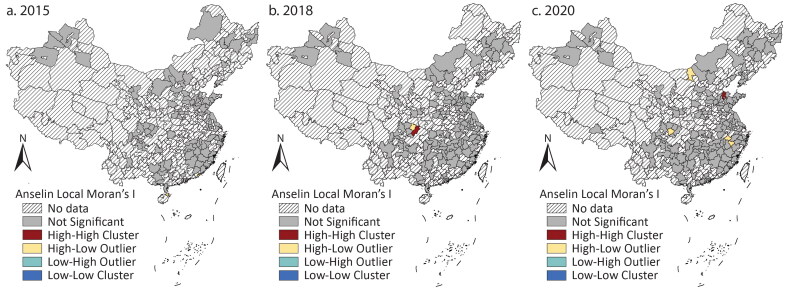
Clusters and outliers of the ratio of local government bond issuance to Chengtou bond issuance in (A) 2015, (B) 2018, and (C) 2020.

Table 3 suggests that there was no sign of clustering in the shift. Anselin’s local Moran’s I hardly identifies any clusters or outliers (Figure 6). These results indicate that although more and more cities changed from *Chengtou* bonds to LGBs, the differences in the issuing amounts of the two bonds were not statistically significant enough to demonstrate a tendency of clustering or dispersion.

**Table 3:** Global Moran’s I and Getis–Ord general G of the ratio of local government bond issuance to Chengtou bond issuance in 2015, 2018, and 2020

	**2015**			**2018**			**2020**		
	**Index**	***Z* score**	***p* value**	**Index**	***Z* score**	***p* value**	**Index**	***Z* score**	***p* value**
Global Moran’s I	−0.020	−0.328	0.743	−0.000	0.482	0.630	−0.000	0.333	0.793
Getis–Ord general G	0.008	−0.889	0.374	0.008	0.417	0.677	0.006	−0.195	0.845

The dynamics indicate that more cities regarded LGBs as the first choice. LGBs were promoted by the central government and backed by the credit of the provincial government. Investors welcomed them as a new low-risk investment option. *Chengtou* bonds were much more scrutinized by investors after 2015. The shift was more evident in the less developed regions.

In most cases, there was no going back to *Chengtou* bonds because the cities kept experiencing a fiscal shortage and could not use LGB capital to repay *Chengtou* bonds. The cities in some eastern provinces could still rely more on *Chengtou* bonds due to their strong economy and fiscal capacity. Cities across the country relied more on LGBs, but *Chengtou* bonds remained one important financing source for infrastructure investment (Z. Li, Wu, and Zhang 2022; Ye et al. 2022). Facing the enduring gap between income and expenditure, cities had to use as many sources as possible to maintain infrastructure investment.

The dynamics complement the understanding of fiscal centralization produced by the dynamics of the total issuance of the two bonds. The total issuance reveals the trend of centralization of local fiscal income and expenditure. The shift shows that fiscal centralization could not restrict local governments’ reliance on *Chengtou* bonds. After separating the debt of LGFPs from local government debt, the central government did not pay as much attention to LGFP debt as it did to local government debt. The accumulation of the debt of local state-owned enterprises could be a problem of local governments but hardly undermined the central government’s policy objective of restricting the financial risk caused by excessive local government debt. On the other hand, the central government left space for local governments to use *Chengtou* bonds. The quotas of LGBs meant that the bonds could not meet the demand of infrastructure finance, and the central and local governments did not want to see a slowdown in growth due to the lack of infrastructure finance.

Local governments have sometimes still issued payment guarantees on behalf of LGFPs secretly after 2015, however. The neglect of the large *Chengtou* bond issuance by the central government and the fact that local governments exaggerated the future income of the infrastructure when applying for LGB quotas led to the financial risk related to “implicit local government debt” newly accumulated. As shown in what follows, such risk was unevenly distributed, and the less developed cities experienced higher risk.

### The Efficiency and Risk

**Figure 7:**
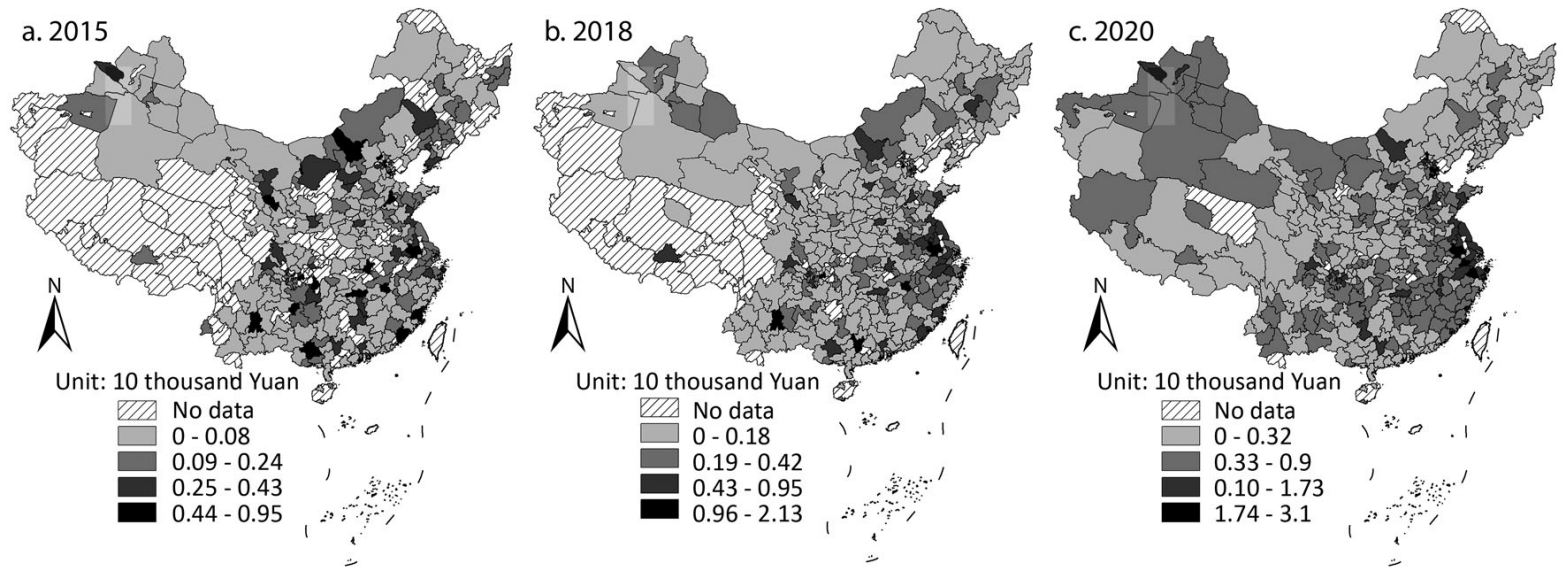
The issuing amounts of local government bonds and Chengtou bonds per person in (A) 2015, (B) 2018, and (C) 2020.

The preceding analysis shows that cities increasingly relied on debt financing for infrastructure finance. Nonetheless, did the less developed cities need the amount of bonds they issued? Figure 7 shows the issuance of LGBs and *Chengtou* bonds per person at the city level. In 2015 and 2018, the cities in the east with better economies had significantly higher values than the less developed ones. The gap narrowed in 2020, though, as many cities in the west and the middle had high values.

**Figure 8:**
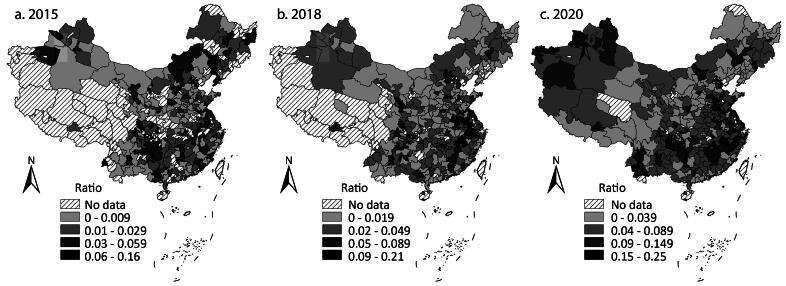
The ratio of the issuing amounts of local government bonds and Chengtou bonds to gross domestic product in (A) 2015, (B) 2018, and (C) 2020.

The less developed cities gradually lost their young population, who went elsewhere for a better life, and those who stayed usually had relatively low living standards (Shen 2013). Some infrastructure here might be unnecessary and could not be used efficiently. Large issuance by the less developed cities imposed high debt ratios on them. Figure 8 shows the ratio of bond issuance to GDP at the city level. Many of the least developed western cities had a higher ratio than the better-off cities in the middle and the east. The situation got worse year by year.

Figures 7 and 8 suggest that the less developed cities did not use *Chengtou* bonds and LGBs efficiently and were imposed disproportionately higher financial risk. The most eye-catching example is a poor county named Dushan in Guizhou Province, one of the least developed provinces in the southwest. Dushan has undertaken many large-scale infrastructure projects since 2012, including a golf course, a university town, a big data center, and a cultural building with a height of 100 m.^9^ Many projects do not match the development of the county. The university town aimed to attract renowned international universities but only two local vocational colleges moved in. The cultural building does not attract many tourists as planned. Most projects are called by the central government “showcase projects” that benefit the resume of local officials but make little contribution to people. Party Secretary Zhili Pan was arrested in 2019, and one of his charges was abusing authority to start many showcase projects.^10^

By 2019, Dushan had borrowed up to 40 billion Yuan mainly through LGFPs. Approximately 14 billion Yuan was counted as local government debt after audits due to payment guarantees and other reasons. Dushan’s annual fiscal income was less than 1 billion Yuan on average in the 2010s and the county could never pay the debt on its own. The municipal, provincial, and even central governments reportedly intervened to avoid any actual default after the county attracted attention nationwide in 2019. The municipal and provincial governments tried to allocate resources within the province to the county and asked for help from the rest of the country.^11^ The central government helped Dushan to produce repayment plans.^12^ Nonetheless, the specific measures and repayment progress have not been disclosed.

Guizhou Province also has difficulties in tackling local government debt. In April 2023, a research institute sponsored by the Guizhou government published a report, suggesting that some cities had severe debt problems, and the province could not fix the problems given the limited fiscal capacity. The report asked the central government for help.^13^ The central government seemed to help, as one of the powerful central state-owned asset management corporations started cooperating with Guizhou in May 2023, although the methods of cooperation were not specified.^14^ Whether the central government could really help remains to be seen.

Analytically, the dynamics and the examples of Dushan and Guizhou reveal that fiscal centralization did not effectively constrain excessive borrowing and consequently could not contain the higher risk experienced by the less developed cities. Practically, the excessive debt of the less developed regions was a burden for the multiscalar governments. The upper level governments needed to use their own resources or those from other places to try to fix the problem, undermining the efficiency and sustainability of local government debt.

## Discussion and Conclusions

This article explores the city-level spatial dynamics of local government debt in China. This mesolevel method furthers the understanding of intergovernmental relations from a disaggregated geographical perspective. The dynamics from 2009 to 2014 were shaped by the central government’s objective of stimulating the economy and the local officials’ pursuit of political career promotion through borrowing as much as possible. The stimulus package led to a large amount of debt, while local governments’ competition imposed large bond issuance on cities across the country. The dynamics of the issuance of *Chengtou* bonds and LGBs after 2015 were shaped by the central government’s objective of restricting debt, and better off cities had larger bond issuance. Local governments’ incentive to compete was secondary to the objective. The dynamics of the shift from *Chengtou* bonds to LGBs show that the central government left space for local governments to finance infrastructure investment through *Chengtou* bonds. The less developed cities had lower efficiency in using *Chengtou* bonds and LGBs and higher financial risk due to limited fiscal capacity.

The dynamics reveal that local government debt in China resulted from changing intergovernmental relations. In the period of LGFPs, the authority over local fiscal income and expenditure was centralized compared to the period of land finance, and the central government intervened more in expenditure than income. In the period of LGBs, local fiscal income and expenditure saw further centralization compared to the period of LGFPs. Fiscal centralization, however, did not constrain the disproportionately higher financial risk experienced by the less developed cities.

These findings, on the one hand, show the importance of the central government in state politics in debt-fueled development (Wu, Zhang, and Liu 2022). The political system in China is centralized, and local governments need and are willing to carry out the central government’s objectives because the political careers of local officials are decided by the upper level decision-makers (He, Zhou, and Huang 2016; Zhou 2016). The dynamics show that fiscal centralization was decided by and served the objectives of the central government rather than local governments.

The central government has many objectives for local governments to implement, but at particular periods of time, the central and local governments might have different priorities. In this situation, the interests of local governments are usually secondary to those of the central government, as the goal of local officials is to align with rather than go against the superiors. In our case, the spatial dynamics of the issuance of *Chengtou* bonds and LGBs were decided by the central government’s objective of restricting financial risk. Even if local officials intended to borrow more, their intention did not significantly affect the dynamics. This echoes Wu, Zhang, and Liu (2022) that local governments need to follow and prioritize the national mandates on rural revitalization, heritage preservation, and others over creating economic benefits in urban redevelopment in China.

On the other hand, the intentions of local governments cannot be ignored. In reality, they sometimes exaggerated the future income of the infrastructure in their applications for LGB quotas and still secretly issued payment guarantees on behalf of LGFPs after 2015, adding “implicit local government debt.” Local governments seemed to implement central policies but their secret behaviors actually to some extent undermined such policies.

The dynamics generate implications for global studies on intergovernmental relations. Examining twelve major countries in developed and emerging markets, Eccleston and Krever (2017) concluded that the world has seen fiscal centralization since the global financial crisis in 2008. Economic stimulus packages commonly used in different countries and the following fiscal consolidation increase intervention by the central government in local fiscal activities. de Mello and Jalles (2020) provided a more detailed analysis using more countries and suggested that during fiscal consolidation, subnational governments could gain increasing bargaining power to negotiate with the central government and influence national policymaking, depending on institutional and political settings.

Our findings revealing the Chinese context echo the emphasis on contextual specificities but do not show Chinese exceptionalism. The situation in China fits the trend of fiscal centralization, although local governments could keep using LGFPs to finance infrastructure construction. The use of LGFPs was hardly an outcome of the central government making compromises with local governments, as the central government’s objective of controlling financial risk was prioritized over the intention of local governments to compete. In reality, local governments indeed caused financial risk. The situation, however, did not alter the conclusions on fiscal centralization and the dominant position of the central government in policymaking.

The importance of the central government and the state as a whole extends the understanding of the findings generated by the mesolevel method. Similar methods are also used in debt studies in the United States, Europe, and other countries (Psycharis, Zoi, and Iliopoulou 2016; Davidson, Lukens, and Ward 2021). These studies talk relatively less about the state but pay more attention to the socioeconomic characteristics of different places in shaping the landscape of debt, as the state might have limited influence over investors. Our findings highlight the multiscalar state and intergovernmental relations when government political and economic objectives at national, regional, and local levels play a more important role in deciding the landscape than place-based characteristics.

The examples of Dushan and Guizhou showed that local officials’ obsession with showcase projects and career promotion wasted money raised through bonds and caused excessive debt. Less developed cities were more vulnerable than the developed ones because they had fewer ways to pay the debt. They might ask for help from others, particularly the central government. Whether such help works and whether the central government could help when more less developed cities and provinces have similar situations remains unknown, however.

## Notes

1 The policy document is at available http://www.npc.gov.cn/zgrdw/npc/lfzt/2014/2000-12/05/content_1875785.htm (accessed October 7, 2023).

2 The introduction to the statistics is based on the official manual for ArcGIS. For global Moran’s I, see https://pro.arcgis.com/en/pro-app/latest/tool-reference/spatial-statistics/spatial-autocorrelation.htm. For Getis–Ord general G, see https://pro.arcgis.com/en/pro-app/latest/tool-reference/spatial-statistics/high-low-clustering.htm. For Anselin’s local Moran’s I, see https://pro.arcgis.com/en/pro-app/latest/tool-reference/spatial-statistics/cluster-and-outlier-analysis-anselin-local-moran-s.htm (accessed October 7, 2023).

3 The Statistical Bureau of China divides the country into four economic regions: the east, the middle, the west, and the northeast. The economy is less and less developed on average from the east, the middle, and the northeast to the west. See https://baike.baidu.com/item/%E4%B8%AD%E5%9B%BD%E5%9B%9B%E5%A4%A7%E7%BB%8F%E6%B5%8E%E5%8C%BA%E5%9F%9F/10619775?fr=ge_ala (accessed October 7, 2023).

4 The introduction to the policy is available at https://www.gov.cn/govweb/gzdt/2009-04/23/content_1293665.htm (accessed October 7, 2023).

5 The news is available at https://www.chinanews.com.cn/cj/gncj/news/2008/11-21/1457768.shtml (accessed October 7, 2023).

6 The views are reported by many authoritative media in China, for example, https://www.cnr.cn/kby/tf/201108/t20110819_508390580.html and https://www.163.com/money/article/8UU670EG00254L5I.html (accessed October 7, 2023).

7 The explanation of the “positive incentive” and the view of the official are reported at https://www.gov.cn/xinwen/2017-04/17/content_5186239.htm (accessed October 7, 2023).

8 The disclosed report is available at https://mp.weixin.qq.com/s?__biz=MzUxNjU4NjY1NA==&mid=2247492145&idx=1&sn=6533d4dac103f134380deda8eb06d4bd&chksm=f9a78fc0ced006d695ac95caf0062d07c3f6c5bb0e79c7fe1197ee03003a456f81cf890b467d&scene=27 (accessed October 7, 2023).

9 The debt problem of Dushan has been widely reported by media on the Internet, for example, see https://baijiahao.baidu.com/s?id=1672149554805953587&wfr=spider&for=pc and https://finance.sina.com.cn/china/gncj/2020-07-14/doc-iivhvpwx5311647.shtml (accessed October 7, 2023).

10 The government is led by the Communist Party of China. The Party Secretary leads as the head of the government.

11 The measures are reported at https://baijiahao.baidu.com/s?id=1672420529842783208&wfr=spider&for=pc (accessed October 7, 2023).

12 The news is reported at https://new.qq.com/rain/a/20211013A0E7UO00 (accessed October 7, 2023).

13 The report was deleted, but was available among different media, such as http://k.sina.com.cn/article_1829577443_6d0d22e30200192qc.html (accessed October 7, 2023).

14 The news is available at https://baijiahao.baidu.com/s?id=1765291233438425944&wfr=spider&for=pc (accessed October 7, 2023).
